# Guided Acoustic Waves in Polymer Rods with Varying Immersion Depth in Liquid

**DOI:** 10.3390/s23249892

**Published:** 2023-12-18

**Authors:** Klaus Lutter, Alexander Backer, Klaus Stefan Drese

**Affiliations:** Institute for Sensor and Actuator Technology, Coburg University of Applied Sciences and Arts, Am Hofbräuhaus 1B, 96450 Coburg, Germany; alexander.backer@hs-coburg.de (A.B.); klaus.drese@hs-coburg.de (K.S.D.)

**Keywords:** guided acoustic waves, piezo transducer, high density polyethylene, L(0,1) mode

## Abstract

Monitoring tanks and vessels play an important part in public infrastructure and several industrial processes. The goal of this work is to propose a new kind of guided acoustic wave sensor for measuring immersion depth. Common sensor types such as pressure sensors and airborne ultrasonic sensors are often limited to non-corrosive media, and can fail to distinguish between the media they reflect on or are submerged in. Motivated by this limitation, we developed a guided acoustic wave sensor made from polyethylene using piezoceramics. In contrast to existing sensors, low-frequency Hanning-windowed sine bursts were used to excite the L(0,1) mode within a solid polyethylene rod. The acoustic velocity within these rods changes with the immersion depth in the surrounding fluid. Thus, it is possible to detect changes in the surrounding media by measuring the time shifts of zero crossings through the rod after being reflected on the opposite end. The change in time of zero crossings is monotonically related to the immersion depth. This relative measurement method can be used in different kinds of liquids, including strong acids or bases.

## 1. Introduction

Monitoring fluids in vessels is an indispensable necessity in various applications in the industrial, medical, and environmental fields. Traditional sensor concepts such as pressure sensors offer high precision and are widely used. While they are independent of the vessel they are submerged in, they are limited to water or other non-corrosive media. In addition, they are influenced by sedimentation at the bottom of a liquid tank. On the other hand, airborne ultrasonic echo sensors may be used for all types of liquids, or even solids; however, foam building up inside the vessel can distort the measurements. To find a solution for all of the aforementioned problems, sensors using guided acoustic waves may present an adequate approach. In previous papers, aluminum rods have been used for the L(0,2) mode as a waveguide [1]. Factors such as the immersion depth have been correlated with the signal energy attenuation because of leakages of waves into the surrounding fluid. Measuring attenuation to determine changes in fluid media is applicable to steel tubes using the aforementioned L(0,2) mode [2]. One sensor concept has used the L(0,1) mode within a nickel and iron wire by measuring the time of flight to determine the surface coverage of the waveguide [3]. All of the mentioned works used metals to propagate acoustic waves, which restricts their use in acidic or basic media. While there are metals resistant to certain acids and bases, these are expensive compared to polyethylene. Here, we present a related approach using polyethylene rods as waveguides. The possibility of exciting acoustic waves within polyethylene and other polymers has been broadly discussed [4,5,6,7,8,9,10,11,12,13,14]. In these papers, dispersion is a crucial feature that needs to be considered when using guided ultrasonic waves, as it distorts wave packets, in turn leading to nonuniform propagation. Therefore, the goal is to minimise both dispersion and attenuation.

## 2. Materials and Methods

### 2.1. Acoustic Wave Propagation in Polyethylene Rods

Acoustic waves in rods propagate in three different types of modes: longitudinal, flexural, and torsional [15,16,17]. The individual modes are influenced differently and in a frequency-dependent manner when liquid is present. A suitable mode and working frequency can be determined on the basis of dispersion diagrams [18,19]. In this work, Disperse simulation software [19] was used to calculate the dispersion diagrams. Because two different solid high-density polyethylene (HD-PE) rods (15 mm and 40 mm in diameter) are investigated in the measurement section of this paper, dispersion graphs of these two configurations are discussed in detail. These rod diameters were chosen because they are common sizes that can easily be ordered in large quantities. The thicker rod has increased stability compared to the smaller one which yields a conceptual advantage in industrial applications. The material parameters of the rods are E=2.2 GPa as the Young modulus, $\rho =960\;kg\;m^{-3}$ as the density, and ν=0.38 as the Poisson ratio, with the following parameters for the water: $\rho =1000\;kg\;m^{-3}$ and $v=1500\;{m\;s}^{-1}$ as the sound velocity.

**Figure 1:**
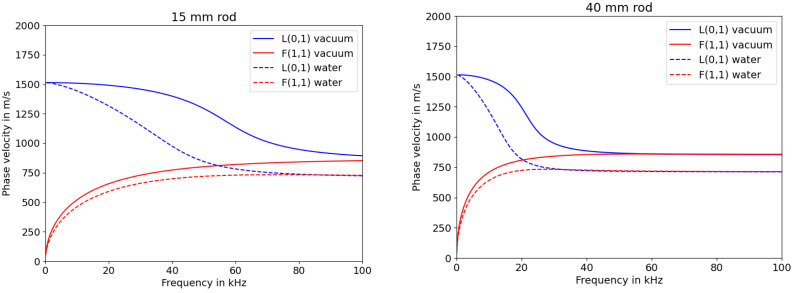
Simulated phase velocities of longitudinal (L) and flexural (F) modes for HD-PE rods with varying diameters.

**Figure 2:**
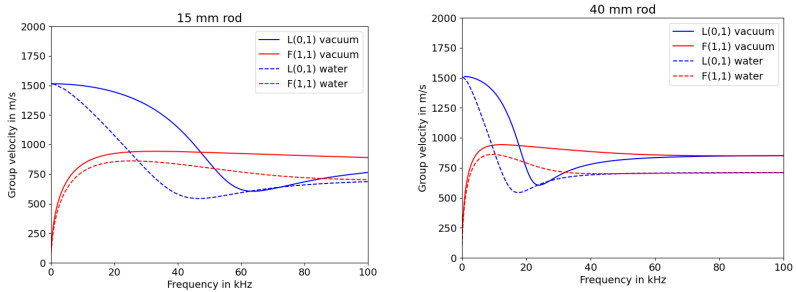
Simulated group velocities of longitudinal (L) and flexural (F) modes for HD-PE rods with varying diameters.

The simulation was set up to have the rod fully immersed in either water or vacuum. It can be seen that there is a difference between the phase velocity depending on the surrounding medium (cp Figure 1). The phase velocities were decreased when submerged in water, leading to the the time shift of the zero crossing positions appearing to be caused by the immersion depth. Another feature to be extracted from these graphs is the desired frequency to be used for exciting the L(0,1) mode. The higher the frequency, the larger the difference in phase velocity due to the surrounding media. Therefore, it was important to find the most adequate frequency that shows a significant difference in phase velocity when submerged in water while analysing the signals and maintaining low dispersion. The dispersion characteristics can be extracted from the graphs in Figure 2.

Sections of the group velocity diagram with high gradients indicate increased dispersion in burst signals, as mentioned in [20]. Looking at the graphs in Figure 2, the group velocity for the 15 mm rod is quite constant for the vacuum scenario at low frequencies, but declines much faster when submerged into water. The 40 mm rod shows a higher gradient even at lower frequencies. This leads to the assumption that the range of frequencies that can be used for measuring the liquid level is significantly smaller for rods with larger diameter, which is comparable to the frequency and plate thickness product for Lamb waves [21].

### 2.2. Signal Excitation

**Figure 3:**
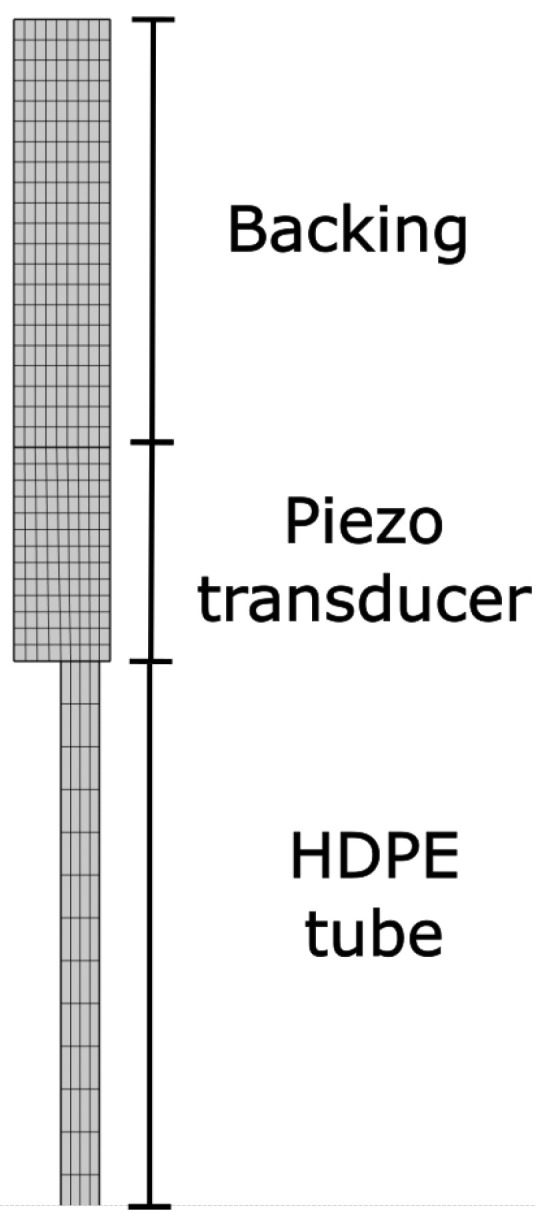
Simulation environment.

Piezoceramics are widely used for excitation of acoustic signals [22]. Depending on their geometry and polarisation, they can be used for longitudinal, torsional, or flexural waves. As the first concept for the approach of a guided acoustic wave sensor for measuring changes in surrounding media was to use polyethylene tubes instead of rods as a way to save both materials and costs, FEM simulations were performed in COMSOL Multiphysics (Version 6.0 [23]) to evaluate the reflected signal of the piezo transducers in multiple configurations. The simluation environment consisted of three different regimes, as can be seen in Figure 3. The model was set up as a two-dimensional rotationally symmetrical model that used the physics interfaces for solid mechanics and electrostatics and the multiphysics interface for piezoelectricity. The HD-PE tube had an outer diameter of 16 mm, a wall thickness of 1.8 mm, and a length of 500 mm. The material parameters for HP-PE matched the ones used in Disperse. To simulate the propagation of acoustic waves within the tube, a five-period Hanning-windowed sine burst with a frequency of 14 kHz was applied to the piezo. The acoustic wave coupled to the HD-PE tube was reflected on the opposite end and coupled back into the piezo transducer. Thus, the change of electric potential was recorded to evaluate the impulse echo propagation of this model. The piezo transducer used the material parameters of the PIC 255 Material by PI Ceramic.

In order to find the ideal piezo geometry, a number of parameter sweep simulations were carried out. To match the shape of the tube, a ring shape was selected. Following parameters were varied:

- inner diameter
- thickness
- backing thickness

**Figure 4:**
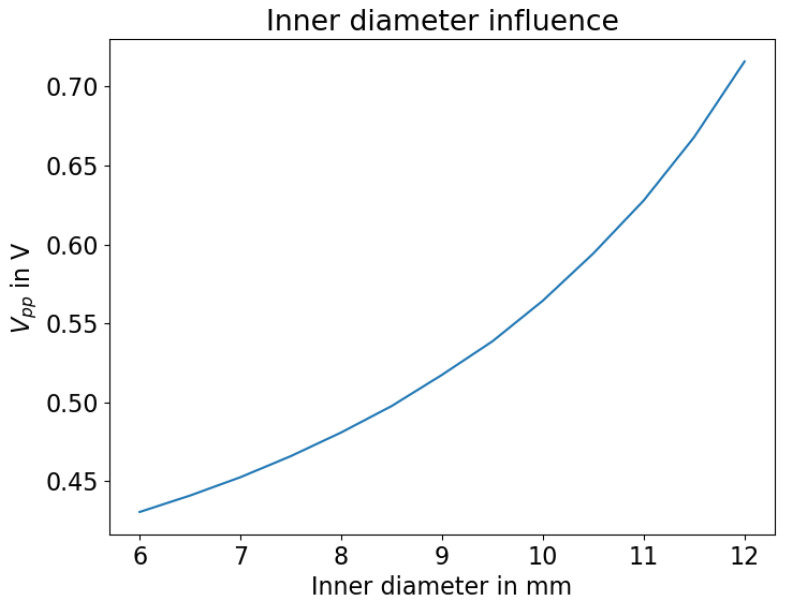
Influence of inner diameter of the piezo ring.

In following figures, the amplitude of the reflected acoustic signal was examined with varying piezo dimensions. The highest detected voltage (peak to peak, $V_{pp}$) of a wavelet is displayed on the y-axis as a target value. Higher amplitudes are preferred in this scenario. On one hand, this increases the signal to noise ratio and thus helps to stabilize any evaluation algorithms. On the other hand, higher amplitudes allow the use of longer rods due to high acoustic attenuation in HD-PE. In Figure 4, the influence of the inner diameter of the ring piezo is displayed. The lower the “wall thickness” of the ring, the higher the reflected amplitude. This can be explained by the decrease in mass that is to be moved by the reflected wave coupling from the HD-PE body into the piezoceramic.

**Figure 5:**
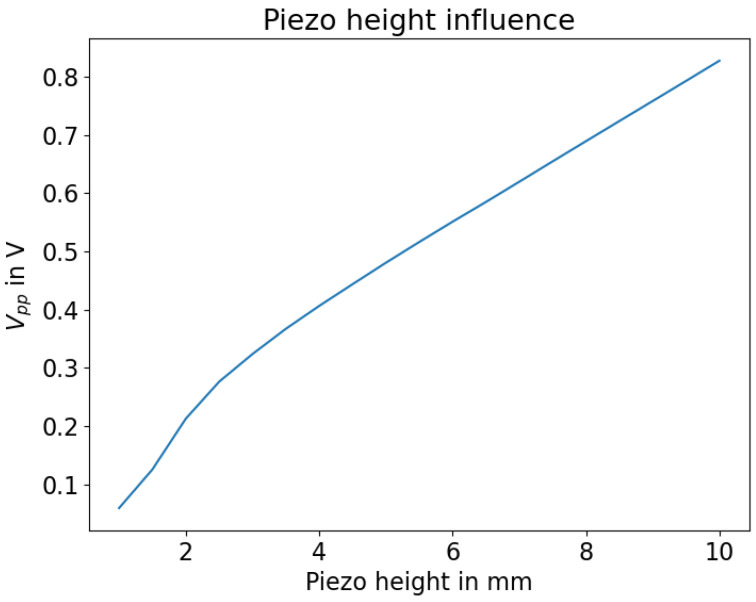
Influence of piezo height.

The influence of the piezo thickness is shown in Figure 5. There, it can be seen that the height positively correlates to the detected signal amplitude. Therefore, thicker piezo rings are to be preferred. The nonlinearity at 1 mm piezo height is caused by the simulation environment. In this parameter configuration, the simulation lattice is too coarse to satisfy the Courant–Friedrichs–Lewy condition [24].

**Figure 6:**
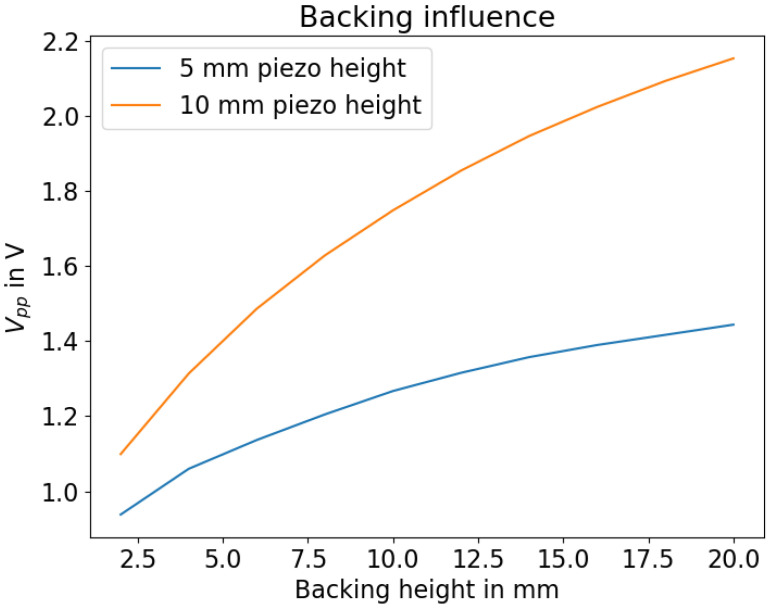
Influence of backing height with different piezo heights.

Backings for piezoelectric transducers were used to increase the amount of energy coupled into the HD-PE body from the piezo transducer. The effect of different backing materials has been discussed in [25,26,27]. In this simulation, stainless steel with E=200 GPa, $\rho =7850\;kg\;m^{-3}$ and ν=0.3 was used. The backing was varied in thickness and compared for two different piezo heights, with the results shown in Figure 6. An increase in backing height leads to increased signal amplitudes. The simulated piezo transducer with a height of 10 mm benefits more significantly from increased backing height.

### 2.3. Experimental Setup

**Figure 7:**
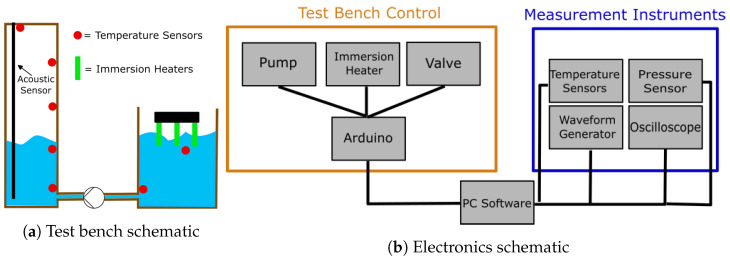
Schematics of the experimental setup.

To measure the change in wave propagation due to varying surrounding media, an experimental setup with a measurement vessel connected to a pump and a surge tank was assembled. This setup schematic is displayed in Figure 7. Six temperature sensors were used to monitor the temperature inside the vessel at four heights near the sensor and in the surge tank. A measurement software was built in Python (3.8, PyQt GUI) to control the valves connecting the two tanks, start and stop the pump, and control the immersion heater. The software was connected to an oscilloscope (LeCroy Waverunner 604 Zi) as well as a waveform generator (Agilent 33500B Series). To be able to measure reflected signals through long solid HD-PE rods, the signal coming from the generator and the reflection picked up by the piezo transducer needed to be amplified. An amplifier with a built-in multiplexer was used to boost the excitation signal by approximately 37 dB and the measured reflection by approximately 30 dB, leading to 190 V peak-to-peak applied to the piezo transducer. The vessel featured a pressure sensor (fluid.iO HD-100) to compare the acoustic measurements to those provided by the water pressure inside the vessel.

### 2.4. Zero Tracing

**Figure 8:**
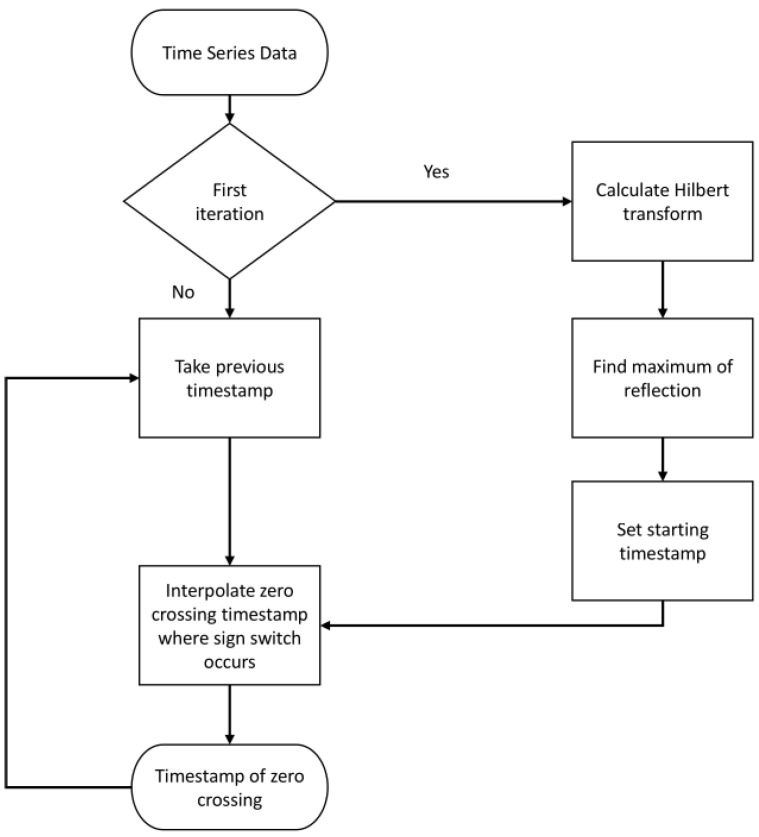
Flow chart of the zero tracing algorithm.

In order to relate the measured signals to the part of the rod immersed in the fluid, it is necessary to track the shift of zero crossing times due to the medium surrounding the rod. This can be achieved by tracing the zero crossings in the reflection of the L(0,1) mode. An algorithm to track these zero crossings was implemented and is explained below. At the beginning, the algorithm determines indices within the time and signal array centered around the maximum of the Hilbert transform of the signal in order to start tracing. To discard the excitation signal, a minimum timestamp needs to be passed. After setting the start indices, the algorithm finds points within the data array where a sign switch happens. The two data points around the sign switch are used to interpolate the time at which the zero crossing occurred. This algorithm is visualized in Figure 8. It can be seen that there is a difference between the rising and falling flanks. Because the excitation or signal frequency is known, phase jumps from one zero crossing to another within the evaluated wavelet can be detected and compensated. Taking the first traced timestamp into account, all following zero crosses can be determined by searching within a range of multiple of signal periods, determined by an input parameter ϵ. These traced signals can then be compared to a reference sensor. This type of tracing algorithm differs from pure time-of-flight measurements. The zero crossing timestamps for minimum and maximum immersion depth need to be known. Every measured timestamp between these values can then be interpolated to display the current immersion depth.

## 3. Results

### 3.1. Piezo Dimensions

**Figure 9:**
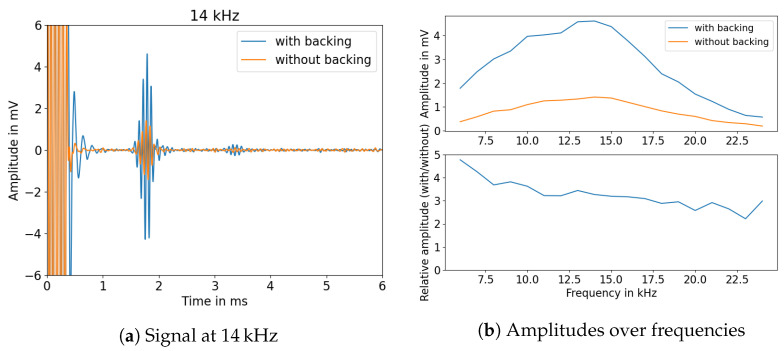
Comparison of signal amplitudes with backing (20 dB amplification).

The simulated piezo parameters were used to order ring shaped piezos (PIC 151, PI Ceramic) according to the simulated dimensions. These piezo transducers were mounted on the solid HD-PE rods and tubes using epoxy glue (UHU plus Schnellfest). A stainless steel backing cylinder was glued to the opposite site of the transducer to improve the energy coupled from the transducer to the HD-PE rod. In order to examine the influence of the backing (stainless steel, height 20 mm) on excited signals, a frequency sweep was measured to compare the maximum of the envelope of the reflected signal in a tube (16 mm diameter, 0.9 m length) without being submerged in water. The resulting signals are visualised in Figure 9.

**Figure 10:**
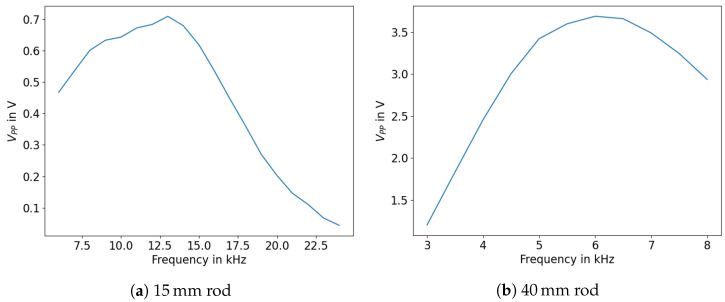
Amplitudes (peak to peak) over various signal frequencies.

As can be seen, the amplitude increases significantly within a HD-PE tube when using a backing for the piezo transducer. In addition, it is possible to extract an ideal frequency to use for highest signal amplitudes. During our experiments, it turned out that a large part of the energy coupled out into the water, meaning that the signals could no longer be evaluated when the tube was immersed more than a few centimeters. Thus, these tubes could not be used for measuring variations in sensor surface coverage. Thus, the experiment was transferred to HD-PE rods, and the ideal excitation frequency was determined similarly. The measurements are shown in Figure 10. It can be seen that the overall signal amplitude is significantly higher for the 40 mm rod. Similar behaviour for amorphous media was investigated in [28].

**Figure 11:**
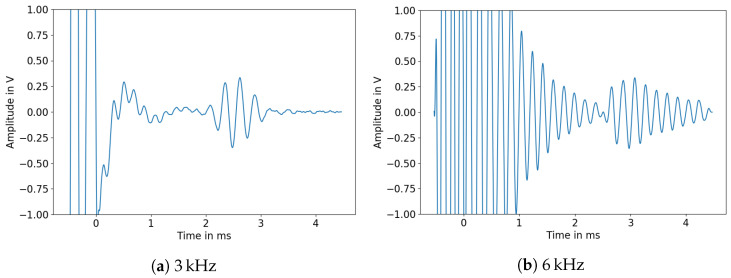
Measured signals of a 40 mm rod (1.7 m length).

The ideal excitation frequency for the solid 15 mm rod was around 13 kHz, whereas the 40 mm rod yielded the largest reflection amplitudes at 6 kHz. High amplitudes are to be preferred due to high attenuation in HD-PE. While these excitation frequencies should be chosen to optimize the signal to noise ratio for measuring liquid levels, the frequencies used in the following sections are predominantly lower, as mounting mechanisms for the rods lead to additional reflections in the signal at these frequencies. The mount applies mechanical stress to the top of the rod near the piezo transducer by clamping onto the rod; one example can be found in Figure 11. There, the signal frequency of 3 kHz was stable enough to use for immersion depth measurements.

### 3.2. Waveforms

**Figure 12:**
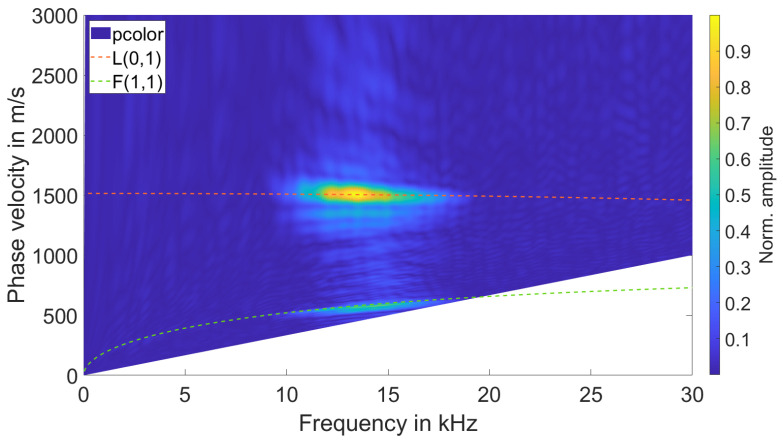
Overlap of dispersion graphs and 2D-FFT transform from laser Doppler vibrometer measurements.

To examine whether the L(0,1) mode could in fact be excited with the aforementioned sensor design, we measured the wave propagation within a 15 mm rod with a laser Doppler vibrometer. We then calculated the 2D-FFT transforms of these signals and compared them to the simulated dispersion graphs. The two graphs overlapped in terms of the excited modes. An example of this procedure can be seen in Figure 12.

**Figure 13:**
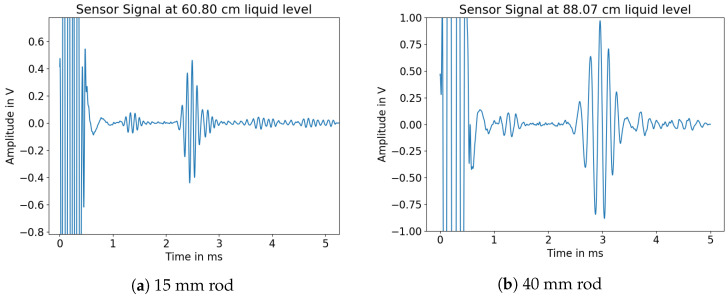
Measured signals of different HD-PE rods.

Looking at the measured waveforms, it was possible to see both the main reflection propagating through the rod as well as the reflection at the fluid surface. One of these signals is shown in Figure 13.

**Figure 14:**
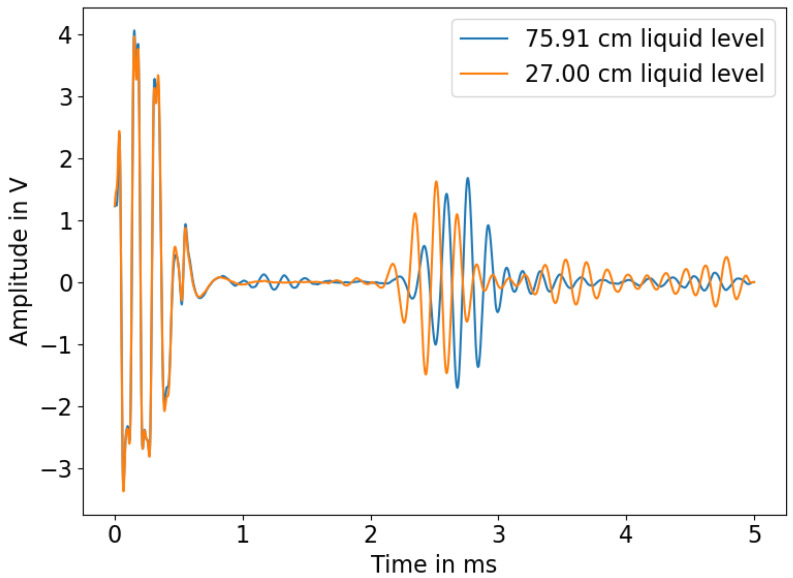
Acoustic signal at two different immersion depths.

It can additionally be seen that the reflected signal coming from the rod is dispersive and that there are multiple wave groups after the reflection of the L(0,1) mode. To further visualize the effect of changing immersion depths on the propagated acoustic signal within the rod, signals with two different immersion depths are shown in Figure 14.

### 3.3. Zero Tracing

**Figure 15:**
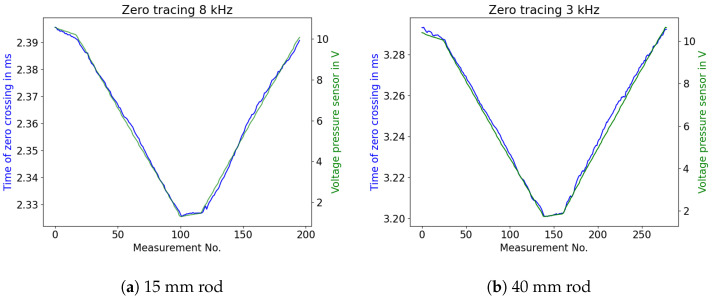
Zero tracing compared to pressure sensor voltages for rods of different diameter.

The algorithm used to trace zero crossings was then applied on signals of two HD-PE rods. Figure 15 shows one measurement cycle of emptying and refilling the vessel. Both the guided acoustic wave sensor and the pressure sensor tracked the falling and rising liquid levels through their respective data points. The x-axis shows the number of measurements that were carried out during one test cycle in the vessel. In contrast to the simulations above, stable detection of reflections of the propagating waves was only possible with much lower excitation frequencies (13 kHz and 6 kHz respectively). This was due to high attenuation within long polyethylene rods (approximately 2 m). In early experiments, small HD-PE probes were examined. There, reflections of excited waves with 50 kHz could still be detected.

**Figure 16:**
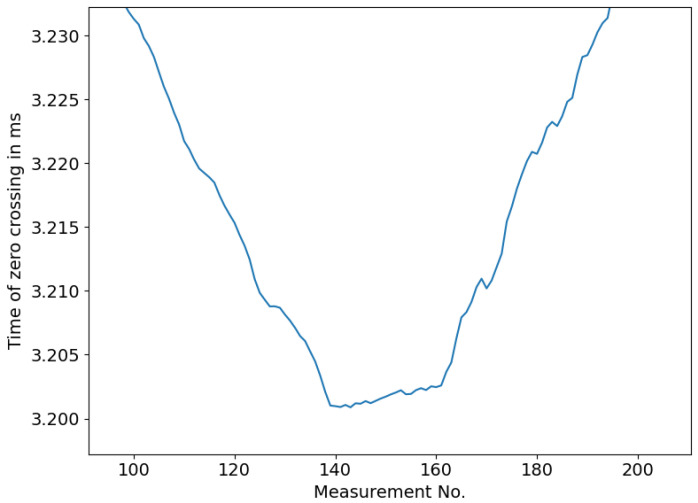
Nonlinear behaviour of traced zero crossing times at low immersion depths, showing a zoomed-in view of Figure 15b.

These traces already show one possible cause of error for using this principle as an immersion depth. At low levels, the reflection of the liquid surface overlaps with the reflection within the rod, leading to nonlinear tracing of the signal’s zero crossings. Figure 16 displays this phenomenon in detail.

### 3.4. Temperature Dependency

**Figure 17:**
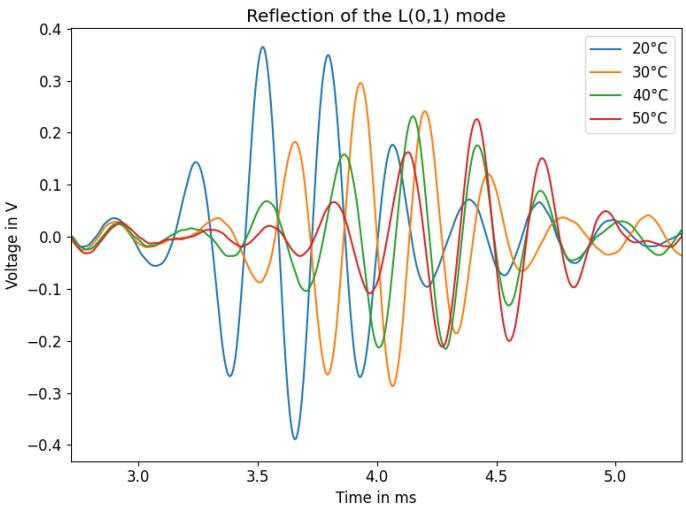
L(0,1) reflection at different temperatures.

Material parameters vary greatly due to changing temperatures. The elastic modulus of HD-PE decreases from 760 MPa to 407 MPa when the temperature increases from 23 °C to 40 °C [29]. Therefore, acoustic wave propagation changes accordingly [30,31]. One sample of a 40 mm rod was tested multiple times for water temperatures of 20 °C, 30 °C, 40 °C, and 50 °C. Two effects were predominantly apparent with temperature change, namely, signal attenuation and dilation of time of flight. With rising temperatures, signal amplitudes decrease and the dilation of the time of flight increases. These phenomena are displayed in Figure 17.

Thus, it is important to compare the overall dilation in the time of flight caused by the change in the surrounding medium to the one caused by temperature. The latter shows a larger amount of dilation. For a 40 mm rod with a three-period 3 kHz sine burst, the average range of zero crossing positions from fully submerged in water to not submerged at all spanned about 80 μs, while the range of the time shift of the wave group due to changes in temperature covered approximately 900 μs. The amplitude of the signal decreased by 42% at 50 °C compared to 20 °C. To compensate these variations in acoustic velocities, it is possible to either set up measurements to thoroughly examine the sensor behaviour within a specific temperature range and calculate compensation curves, or to try to excite additional acoustic modes in order to use multiple propagation times to create a compensation mechanism [32].

## 4. Discussion

In this work, we present a novel approach to monitor changing fluid media in vessels using polyethylene rods. Compared to similar articles, we used lower frequencies to excite the acoustic waves due to attenuation and dispersion within amorphous media. Even though the concept worked in a laboratory environment, there are significant challenges that need to be overcome before this technique can be used in industrial applications. First, because the dilation caused by temperature exceeds the measured effect of the changing fluid media, the temperature drift needs to be thoroughly examined in order to calculate a compensation curve. The mounting mechanism poses a challenge as well, as reflections or multiples thereof can interfere with the desired pulse echo. In several of our experiments this led to the reflection at the surface not being traceable. Another aspect to be investigated is the possibility of amplifying both the excited signal and the received signal, as plastics such as polyethylene have much higher attenuation compared to most common metals used for guided acoustic waves. To conclude, this approach might bring advantages to certain industrial applications where using metal rods as waveguides or different sensors is generally not possible.

## Data Availability

The data presented in this study are available on request from the corresponding author. The data are not publicly available due to secrecy.

## Abbreviations

The following abbreviations are used in this manuscript:

HD-PE	High Density Polyethylene

## References

[1] Subhash N.N., Krishnan B. (2014). Fluid level sensing using ultrasonic waveguides. Insight.

[2] Dhayalan R., Saravanan S., Manivannan S., Purna Chandra Rao B. (2020). Developement of ultrasonic waveguide sensor for liquid level measurement in loop system. Electron. Lett..

[3] Royer D., Levin L., Legras O. (1993). A Liquid Level Sensor Using the Absorption of Guided Acoustic Waves. IEEE Trans. Ultrason. Ferroelectr. Freq. Control.

[4] Shah J., El-Hawwat S., Wang H. (2023). Guided Wave Ultrasonic Testing for Crack Detection in Polyethylene Pipes: Laboratory Experiments and Numerical Modeling. Sensors.

[5] Sinha M., Buckley D.J. (2007). Acoustic Properties of Polymers. Physical Properties of Polymers Handbook.

[6] Ozaki R., Kadowaki K. (2020). Analysis of Attenuation and Dispersion of Acoustic Waves in Low-Density Polyethylene. IEEE Trans. Dielectr. Electr. Insul..

[7] Qi G., Li Y., Ding N. Measurement of Acoustic Basic Parameters of Polyethylene Pipe. Proceedings of the IOP Conference Series: Materials Science and Engineering.

[8] Pylaev A.E., Kostikova E.A., Yurkov A.L. (2018). Velocity and Attenuation of Acoustic Waves in Polymers and Polymer Composites. Polym. Sci. Ser. D.

[9] Mazeika L., Sliteris R., Vladisauskas A. (2011). Measurement of velocity and attenuation for ultrasonic longitudinal waves in the polyethylene samples. Ultragarsas J..

[10] Jordan J.L., Rowl R.L., Greenhall J., Moss E.K., Huber R.C., Willis E.C., Hrubiak R., Kenney-Benson C., Bartram B., Sturtevant B.T. (2021). Elastic properties of polyethylene from high pressure sound speed measurements. Polymer.

[11] Egerton J.S., Lowe M.J.S., Huthwaite P., Halai H.V. (2017). Ultrasonic attenuation and phase velocity of high-density polyethylene pipe material. J. Acoust. Soc. Am..

[12] Rautenberg J., Bause F., Henning B. Utilizing guided acoustic waves to measure dispersive material properties of polymers. Proceedings of the SENSOR 2015.

[13] Kwun H., Bartels K.A., Dynes C. (1999). Dispersion of longitudinal waves propagating in liquid-filled cylindrical shells. J. Acoust. Soc. Am..

[14] Wilcox P.D. (2003). A rapid signal processing technique to remove the effect of dispersion from guided wave signals. IEEE Trans. Ultrason. Ferroelectr. Freq. Control.

[15] Gazis D.C. (1959). Three-Dimensional Investigation of the Propagation of Waves in Hollow Circular Cylinders. I. Analytical Foundation. J. Acoust. Soc. Am..

[16] Gazis D.C. (1959). Three-Dimensional Investigation of the Propagation of Waves in Hollow Circular Cylinders. II. Numerical Results. J. Acoust. Soc. Am..

[17] Silk M.G., Bainton K.F. (1979). The propagation in metal tubing of ultrasonic wave modes equivalent to Lamb waves. Ultrasonics.

[18] The Dispersion Calculator: An Open Source Software for Calculating Dispersion Curves and Mode Shapes of Guided Waves. https://www.dlr.de/zlp/en/desktopdefault.aspx/tabid-14332/24874_read-61142/.

[19] Pavlakovic B., Lowe M., Alleyne D., Cawley P. (1997). Disperse: A General Purpose Program for Creating Dispersion Curves. Review of Progress in Quantitative Nondestructive Evaluation: Volume 16.

[20] Wilcox P. (1999). Long Range Lamb Wave Inspection: The Effect of Dispersion and Modal Selectivity. Review of Progress in Quantitative Nondestructive Evaluation: Volume 18A–18B.

[21] Lowe M.J.S. (2001). WAVE PROPAGATION|Guided Waves in Structures. Encyclopedia of Vibration.

[22] Duquennnoy M., Ouaftouh M., Qian M.L., Jenot F., Ourak M. (2001). Ultrasonic characterization of residual stresses in steel rods using a laser line source and piezoelectric transducers. NDT E Int..

[23] Comsol Multiphysics: Simulate Real-World Designs, Devices, and Processes with Multiphysics Software from COMSOL. www.comsol.com.

[24] Courant R., Friedrichs K., Lewy H. (1967). On the Partial Difference Equations of Mathematical Physics. IBM J. Res. Dev..

[25] Moreno E., Pereira W., von Kruger M.A., Leija L., Ramos A. (2022). FEM modeling and simulation of broadband ultrasonic transducers with randomized inhomogeneous backing material. arXiv.

[26] Kossoff G. (1966). The Effects of Backing and Matching on the Performance of Piezoelectric Ceramic Transducers. IEEE Trans. Sonics Ultrason..

[27] Subki M.S.H.M., Ahmad K.A., Osman M.K., Boudville R., Yahya S.Z., Rahman M.F.A., Hussain Z. Characterization of Backing Layer Piezoelectric Ultrasonic Transducers for Underwater Communication. Proceedings of the IEEE International Conference on Control System, Computing and Engineering.

[28] Benatar A., Rittel D., Yarin A.L. (2003). A Theoretical and experimental analysis of longitudinal wave propagation in cylindrical viscoelastic rods. J. Mech. Phys. Solids.

[29] Nomura R., Yoneyama K., Ogasawara F., Ueno M., Okuda Y., Yamanaka A. (2003). Temperature Dependence of Sound Velocity in High-Strength Fiber-Reinforced Plastics. Jpn. J. Appl. Phys..

[30] Wada Y., Yamamoto K. (1956). Temperature Dependence of Velocity and Attenuation of Ultrasonic Waves in High Polymers. J. Phys. Soc. Jpn..

[31] Merah N., Saghir F., Khan Z., Bazoune A. (2006). Effect of temperature on tensile properties of HDPE pipe material. Plast. Rubber Compos..

[32] Landskron J., Dötzer F., Benkert A., Mayle M., Drese K.S. (2022). Acoustic Limescale Layer and Temperature Measurement in Ultrasonic Flow Meters. Sensors.

